# Emotional profile of athletes before competition: contributions for perceived stress, cognitive appraisal and coping strategies

**DOI:** 10.3389/fspor.2025.1636826

**Published:** 2025-07-30

**Authors:** José Miguel Nogueira, Catarina Morais, Paul Mansell, A. Rui Gomes

**Affiliations:** ^1^ School of Psychology, University of Minho, Braga, Portugal; ^2^ Faculty of Education and Psychology, Universidade Católica Portuguesa, Porto, Portugal; ^3^ School of Health, Education, Policing and Sciences, University of Staffordshire, Stoke-on-Trent, United Kingdom

**Keywords:** arousal (of emotion), emotional profile, threat, challenge, humor, denial, active coping, emotional support

## Abstract

**Introduction:**

Understanding athletes' emotional experience prior to competition is crucial for examining their adaptation to stress. Earlier research suggested anxiety impaired performance by disrupting tasks like information processing, attention, and concentration—leading to increased stress and perceived threat. Over time, focus shifted toward understanding how athletes' interpretations of anxiety could influence performance positively. This led to broader research into other emotions typically considered “negative” (e.g., anger, dejection) and “positive” (e.g., excitement, happiness). However, how these emotions influence performance and interact with intensity has been under-studied.

**Methods:**

A total of 383 elite athletes completed a questionnaire 24–48 h before a major competition, assessing overall stress, emotional intensity (excitement, happiness, anxiety, anger, dejection), emotional direction, cognitive appraisal, and coping strategies.

**Results:**

A cluster analysis based on emotion intensity and direction identified three athlete profiles: “Emotionally Balanced” (moderate intensity), “Facilitating Arousal Profile” (mixed intensity, all emotions viewed as performance-enhancing), and “Low Arousal Profile” (low emotional intensity). Despite differing emotional profiles, athletes reported similar stress levels before competition. However, those in the “Facilitating Arousal Profile” reported greater challenge appraisals, perceived control, and use of adaptive coping strategies compared to others.

**Discussion:**

These findings suggest that not just emotional intensity but also the perceived impact of emotions plays a key role in performance. These results have important implications for psychological interventions, emphasizing the need to consider both how emotions are experienced and how they are interpreted in the context of competition.

## Introduction

Research into the emotional experience of athletes in a competitive context is a topic that has generated great interest in sport, contributing to a deeper understanding of the relationship between emotions and sporting performance [e.g., (1)] and adaptation to stressful situations [e.g., (2, 3)]. Previously, research converged on the idea that anxiety led to a deterioration in performance by interfering with certain tasks (e.g., information processing; attention; concentration), leading to the experience of higher levels of stress and, consequently, an increase in the perception of threat (4). Gradually, the focus shifted to understanding that the way athletes viewed competition could lead them to attribute a potentially beneficial effect on performance to anxiety states [e.g., (5)]. For example, a systematic review reported that anger and anxiety were emotions that although potentially damaging to performance, could also positively influence performance if athletes learned to harness these emotions (4). This has led to the study of other emotions, whether typically considered as negative (e.g., anger, dejection) or positive (e.g., excitement, joy) [e.g., (6)]. Taken together, it appears that the intensity and the direction (perception of usefulness) of emotions both play a role in athletic performance (7).

## Cognitive, motivational and relational theory

The study of emotions in sport has advanced with the contributions made by Lazarus’ transactional, cognitive, motivational and relational (CMR) model (1991, 2000a), which has been applied to the sports context (7). Indeed, support for the CMR model in high-performing athletes has been reported by Doron and Martinent (8). In CMR theory, emotion is understood as a reaction to the continuous relationships that are established between the athlete and the environment (9). Simply put, the personal characteristics of the athlete combined with those of a particular situation will determine how they appraise the situation and their emotional outcomes (10). This process is dynamic meaning that individuals are required to adjust to the demands of the context (9). Resultingly, given the known associations between emotions, cognitive appraisals, and outcomes such as performance and mental health, evolving our understanding of how such factors relate is important in developing knowledge as to how these processes can be optimized to best support athletes (8, 11).

### Cognitive appraisal

Within CMR theory, cognitive appraisal consists of primary and secondary appraisals, which inform the coping process. Primary appraisal is an evaluation of the significance of an event including anticipated loss or gain, while secondary appraisal refers to an individual considering the coping strategies that they have available to them (9). Building on the stress theory put forth by Lazarus, The Theory of Challenge and Threat States in Athletes outlines that when athletes interpret anxiety as beneficial to performance, this can contribute towards evincing a challenge state [TCTSA; (12)]. In turn, this makes performing well more likely (13). Within this theory, consideration was given to the role and interpretation of emotions as either facilitative or debilitative for performance, and other antecedents such as perceptions of control, self-efficacy, and approach/avoidance motivation focus were recognised. More recently, the role of social support and trait dispositions were given greater credence in determining whether athletes experience challenge or threat states (14).

### Emotions

Emotions are defined as “an organized psychophysiological reaction to ongoing relationships with the environment, most often, but not always, interpersonal or social” [(2), p. 230]. Emotions are said to emerge from an initial assessment that athletes make about a meaningful performance situation through cognitive evaluation (11). They are dynamic and can change throughout the stress process and in response to an outcome, such as a result (15). As proposed by the TCTSA (12), the way in which an athlete appraises a motivated performance situation will result in a challenge or threat state. If the perception of threat occurs when the situation is evaluated as potentially negative, this will likely be unhelpful for performance. In contrast, when a challenge state is evinced, the athlete will likely experience greater self-efficacy, perceptions of control, and an approach motivation focus (12). Generally speaking, research has shown that threat is related to negative emotions and challenge to positive emotions, arguing that challenge states lead to the expectation of higher performance (1, 14). Such relationships were evidenced in studies with golfers—those who experienced greater threat were more likely to report higher anxiety and interpret anxiety responses as debilitative. In contrast, those who experienced greater challenge were more likely to report fewer negative emotions, more positive emotions, and to interpret anxiety as facilitative to performance (16).

According to the Directional Interpretation Hypothesis (17), emotions can be examined in terms of their intensity or their direction (i.e., whether we think they are facilitative or debilitative to performance). Studies support this assertion noting that anxiety direction is a stronger predictor of performance than anxiety intensity (18) and that facilitative interpretation of pre-competition experience fewer negative emotions (19). Regarding specific emotions, although there is the suggestion that anxiety can undermine performance [e.g., in rugby players; (20)], the relationship between anxiety and performance may be more nuanced. Although individuals may not enjoy feelings of anxiety, such feelings can functionally serve as a reminder of the importance of an event (21, 22). Research has also presented different understandings of the role that anger can play in adaptation to stressful situations. While it can produce benefits for performance and is associated, in some studies, with challenge appraisals [e.g., (23)], anger is also associated with reduced control over the situation (24), worse performance (20), and use of less functional coping strategies, such as passive emotional regulation (25). When referring to the role of dejection to influence performance, studies and theoretical prepositions are clear about the maladaptive nature of dejection as it is an emotion that elicits reduced mobilization for action (7). Dejection is also associated with avoidance coping strategies (26) which may not be conducive to athletic performance. For the emotions of excitement and joy, these are understood to be more beneficial for performance, as they are usually associated with more favorable modes of cognitive evaluation and more effective coping strategies [e.g., (27)].

### Coping

Lazarus (7) explains that coping refers to our ability to manage our emotions, and like cognitive appraisal and emotions, they are dynamic and are influenced by factors such as the environment and feedback. As part of the cognitive appraisal process, an analysis is made of the coping options available. In addition to the perception of coping, which is related to the perceived ability to combat adversity, it also includes the perception of control, linked to the understanding that it is up to the athlete to manage and reduce stress levels (2). Research suggests that coping skills and the perception of greater control over the situation are associated with the attribution of a more positive effect of emotions on performance and the use of more functional coping strategies (1, 25).

The aim of the study was to analyze whether differences in intensity and direction of athletes’ emotional experiences in relation to performance are associated with cognitive appraisals and coping. It was hypothesized that athletes with moderate levels of intensity and who perceived emotions as facilitative to performance would have more adaptive patterns of cognitive appraisal and coping.

## Method

### Procedure

The study was approved by the Ethics Committee of the University of Minho (reference 026/2014) and followed a cross-sectional design. Clubs competing at the elite level were contacted to participate in the study. Then, athletes from these clubs were contacted and provided with opportunity to give informed consent. In the case of under aged athletes, consent was also obtained from parents/legal guardians. The response rate was of 92% (383 out of 415 athletes agreed to participate). Pen-and-pencil questionnaires were completed in the presence of the main researcher. Using a critical incident methodology, participants completed the questionnaire 24h–48 h before an upcoming competition. The final stages of the season, in which championships and knockout stages of national cups were in question, were selected to capture the most potential stressful competitions.

### Participants

In total, 383 athletes (229 males and 154 females), aged between 14 and 37 years old (*M* = 22.85; *SD* = 5.35) competing at top level of their sport^1^ agreed to participate in the study. The sample included athletes from individual (*n* = 157; 41%) and team sports (*n* = 226; 59%). Individual sports included 105 swimmers and 52 runners, while team sports athletes included 125 handball and 101 volleyball players. In terms of experience, it ranged from 3 to 27 years (*M* = 11.65, *SD* = 4.93), and 72% of athletes had been a national champion at least once, and more than half (55%) had already represented their national team at least once (average of 17 international caps). During the season in which data was collected, 61% athletes were in a position of winning the national championship and 5% were competing at European or World levels.

### Measures

#### Emotions

The Sport Emotional Questionnaire [SEQ; (28)] was used to assess athletes’ feelings regarding the upcoming competition. A set of 22 emotions were displayed, and athletes were asked to report the intensity (0 = *Not at all*; 4 = *Extremely*) and direction of impact on performance (−3 = *Very negative to performance*; 0 = *Indifferent to performance*; +3 = *Very positive to performance*). The emotions assessed included (1) excitement (*α*_intensity_ = .86; *α*_direction_ = .88), (2) happiness (*α*_intensity_ = .93; *α*_direction_ = .87), (3) anxiety (*α*_intensity_ = .86; *α*_direction_ = .80), (4) dejection (*α*_intensity_ = .88; *α*_direction_ = .91), and (e) anger (*α*_intensity_ = .69; *α*_direction_ = .72). Participants’ responses were averaged, so higher values indicate greater intensity and a more positive effect of emotion on performance for each athlete. The psychometric properties of this instrument for this study were acceptable for the intensity evaluation [*χ*^2^ (198) = 459.74, *p* < .001; RMSEA = .059, 90% C.I. [.052;.066]; CFI = .948; NFI = .913; TLI = .939] and direction evaluation [*χ*^2^ (199) = 460.55, *p* < .001; RMSEA = .059, 90% C.I. [.052;.066]; CFI = .944; NFI = .906; TLI = .935].

#### Cognitive appraisal

Using the Primary and Secondary Cognitive Appraisal Scale [PSCAS (29);], athletes were primed to think about the upcoming competition and evaluate their primary cognitive appraisal (including challenge perception, *α* = .61; and threat perception, *α* = .64) and secondary cognitive appraisal (including coping perception, *α* = .84; and control perception, *α* = .86). Participants assessed each of the 12 items, 3 per dimension, using a seven-point Likert scale (ex: *1* = *Is not threatening to me*; 7 = *Is very threatening to me*). Their responses were averaged to compute a dimension score, with higher levels indicating higher levels of the dimension. The psychometric properties of this instrument for this study were acceptable [*χ*^2^ (48) = 165.85, *p* < .001; RMSEA = .080, 90% C.I. [.067; .094]; CFI = .928; NFI = .903; TLI = 0.901].

#### Coping

The Reduced Coping Inventory [Coping-R (30); in press] was used. First, athletes were asked to indicate their overall stress level (*1* *=* *low stress, 5* *=* *high stress*) caused by the situation of “not achieving the desired performance in the upcoming competition”. Then, they rated the use (*1* *=* *I will never use it, 5* *=* *I will use it often*) of coping strategies to deal with the situation, namely: active coping (*α* = .81), humor (*α* = .83), denial (*α* = .65), and emotional support (*α* = .90). The psychometric properties of this instrument for this study were acceptable [*χ*^2^ (98) = 197.064, *p* < .001; RMSEA = .051, 90% C.I. [.041; .062]; CFI = .960; NFI = .925; TLI = .952; CMIN = 2.011].

## Results

### Data analysis

For all data analyses, SPSS (Version 26.0) was used. Before testing the goals of this study, we checked the distribution of the results and found that followed a normal distribution [*sk* ≤ |3| and *ku* ≤ |10|; cf (31)]. Then, to create participants’ emotional profiles, cluster analysis was conducted, using K-Means method due to sample size and the fact that all variables included to form profiles (intensity and perceived influence on performance of excitement, happiness, anxiety, dejection and anger) were continuous variables. Prior to the analyses, all variables were standardized.

### Athletes’ emotional profile

A solution of three profiles were found to best account for a balance between number of athletes per profile and profile differentiation. Table 1 summarizes the three profiles. Profile A is characterized by moderate intensity of emotions and, in terms of direction, typically “positive” emotions are perceived as facilitating performance and typically “negative” emotions perceived as debilitating performance. Thus, athletes in this cluster seem to emphasize emotional moderation—athletes in the Profile A have been named ‘Emotionally Balanced’. Profile B included athletes who feel intense excitement and happiness prior to competitions, average anxiety and low dejection and anger. Regardless of the intensity, these athletes tend to perceive all emotions as facilitating performance. Thus, this profile has been named ‘Facilitating Arousal Profile’. Finally, athletes from Profile C have similar perceptions regarding the role of emotions to performance as athletes “Emotionally Balanced”, but athletes on this cluster generally report low intensity of emotions prior to competitions. Thus, this profile has been categorized as ‘Low Arousal Profile’.

**Table 1:** Athletes’ emotional profile characterization.

Dependent variables	Profile A ‘Emotionally balanced’ (*n* = 42)		Profile B ‘Facilitating arousal Profile’ (*n* = 149)		Profile C ‘Low arousal profile’ (*n* = 192)	
	*M* (*SD*)	Classification	*M* (*SD*)	Classification	*M* (*SD*)	Classification
Intensity of emotions [0; 4]						
Excitement	2.02 (0.75)	Medium	2.84 (0.55)	High	1.88 (0.67)	Medium
Happiness	1.36 (1.12)	Low	3.23 (0.64)	High	2.31 (0.92)	Medium
Anxiety	2.12 (1.05)	Medium	1.80 (0.83)	Medium	1.52 (0.85)	Low
Dejection	1.81 (0.86)	Medium	0.09 (0.23)	Low	0.23 (0.34)	Low
Anger	1.95 (0.93)	Medium	0.18 (0.43)	Low	0.17 (0.31)	Low
Direction of emotions [−3; 3]						
Excitement	0.96 (1.07)	Facilitating	1.92 (0.55)	Facilitating	0.93 (0.79)	Facilitating
Happiness	0.63 (1.11)	Facilitating	2.13 (0.74)	Facilitating	1.25 (0.87)	Facilitating
Anxiety	−0.05 (0.99)	Neutral	0.61 (1.01)	Facilitating	−0.17 (0.78)	Neutral
Dejection	−0.30 (1.10)	Neutral	0.91 (1.36)	Facilitating	−0.44 (0.90)	Neutral
Anger	0.12 (1.20)	Neutral	1.18 (1.20)	Facilitating	−0.36(0.87)	Neutral

Classification for intensity: (1) *low* when values between 0 and 1.5; (2) *medium* when between 1.6–2.5; (3) *high* when >2.5. Classification for direction (perceived effect of emotion on performance): (1) *debilitating* when values between −3 and −0.6; (2) *neutral* when between −0.5 and 0.5; (3) *facilitating* to performance when >0.5.

One-Way ANOVAs and Chi-square tests were conducted to assert whether athletes belonging to different emotional profiles had different sociodemographic characteristics. There were no differences in terms of age [*F*(2,375) = 0.31, *p* = .738], international caps [*F*(2,375) = 0.05, *p* = .956], years of practice [*F*(2,375) = 1.96, *p* = .143], nor gender [*χ*^2^(2) = 5.75, *p* = .056]. However, there were differences regarding type of sport [*χ*^2^(2) = 6.64, *p* = .036] and competitive level [*χ*^2^(6) = 29.29, *p* < .001]. Specifically, the “Emotionally Balanced” group included proportionally more athletes from team sports (vs. individual sports) and less athletes competing to win national championships (vs. in a position of not fighting for the national title) than the remaining groups.

### Stress, cognitive appraisal, and coping among different emotional profiles

ANCOVAs and MANCOVAs were conducted to compare athletes from different emotional profiles on their overall stress, cognitive appraisal, and coping. Type of sport and competitive level were included as covariates, and *post-hoc* analyses were conducted with Bonferroni correction. Table 2 summarizes the results.

**Table 2:** ANCOVA results, means (SD) for all profiles.

Dependent variables	Profile A ‘Emotionally balanced’ (*n* = 42)	Profile B ‘Facilitating arousal profile’ (*n* = 149)	Profile C ‘Low arousal profile’ (*n* = 192)	Simple main effects *F*(2,382), *p*
Overall stress	3.60 (1.23)	3.44 (1.07)	3.24 (1.09)	*F* = 2.09, *p* = .125
Cognitive appraisal [*λ* = .78, *F*(4, 375) = 12.39, *p* *<* .001, *ηp*^2^ = .11]				
Threat	5.06 (1.33)^a^	4.53 (1.16)^a,b^	4.30 (1.12)^b^	*F* = 6.35, *p* = .002, *ηp*^2^ = .03
Challenge	5.79 (1.08)^a^	6.44 (0.60)^b^	5.96 (0.81)^a,c^	*F* = 21.11, *p* < .001, *ηp*^2^ = .10
Coping	5.23 (1.18)^a^	6.01 (0.71)^b^	5.40 (0.92)^a,c^	*F* = 28.32, *p* < .001, *ηp*^2^ = .13
Control	4.13 (1.50)^a^	5.20 (1.14)^b^	4.76 (1.36)^a,c^	*F* = 12.48, *p* < .001, *ηp*^2^ = .06
Coping Strategies [λ = .82, *F*(4, 375) = 9.50, *p* *<* .001, *ηp*^2^ = .09]				
Active coping	3.75 (0.72)^a^	4.06 (0.74)^b^	3.59 (0.78)^a,c^	*F* = 16.31, *p* < .001, *ηp*^2^ = .08
Emotional support	3.01 (0.94)^a^	3.20 (0.96)^a,b^	2.83 (0.86)^a,c^	*F* = 8.11, *p* < .001, *ηp*^2^ = .04
Humor	2.15 (0.94)	1.84 (0.89)	1,97 (0.97)	*F* = 2.55, *p* = .079
Denial	2.37 (0.81)^a^	1.65 (0.57)^b^	1.74 (0.65)^b,c^	*F* = 19.99, *p* < .001, *ηp*^2^ = .10

Letters ^a,b,c^report *post-hoc* results.

Regardless of their emotional profile, athletes reported similar overall stress prior to the competition. However, athletes from Profile B (‘Facilitating Arousal Profile’, who tend to perceive emotions as facilitating performance), reported higher levels of challenge, coping and control, and also reported to be more likely to use active coping and emotional support (adaptive coping strategies) than ‘Emotionally Balanced’ and ‘Low Arousal Profile’ athletes. Moreover, ‘Emotionally Balanced’ (Profile A) athletes reported higher threat and use of denial than athletes belonging to the other profiles. There were no differences regarding the use of humor as a coping strategy among athletes.

## Discussion

The aim of the present study was to analyze whether differences in intensity and direction of athletes’ emotional experiences in relation to performance are associated with cognitive appraisals and coping. Using cluster analysis based on the intensity and direction of emotions prior to performance, three profiles were created. The profiles were then analyzed in relation to how they may relate to cognitive appraisals and coping strategies.

### Emotional profiles: which is most helpful for athletes?

Overall, none of the profiles can be said to be outright unhelpful for performance. Although we observed a variety of responses in terms of emotion intensity, emotions that are typically considered to be negative were not reported as high in any profile. For example, dejection was reported as low intensity in the ‘Facilitating Arousal’ and ‘Low Arousal’ profiles, and medium intensity in the ‘Emotionally Balanced’ profile. Concurrently, intensity of happiness was also low in the ‘Emotionally Balanced’ profile. However, in the ‘Emotionally Balanced’ profile, none of the emotions were perceived as debilitative towards performance. This is important if we consider that it is often the interpretation of emotions rather than the intensity which predicts performance (18). With this in mind, the ‘Facilitating Arousal’ profile may appear to be the *most* adaptive for performance. Athletes in this cluster were able to view all emotions as facilitative for performance—a trait that can enable more adaptive responses to competition (12). To illustrate, athletes in the ‘Facilitating Arousal’ profile reported a medium intensity of anxiety but reported viewing this as facilitative for performance, perhaps seeing such emotions as a sign of readiness to perform well (22).

### Emotional profiles: relationship with appraisals and coping

Emotions, the interpretation of their functionality for performance, cognitive appraisals, and coping [e.g., (9, 11)] are inextricably linked as part of a dynamic stress process. Although none of the profiles appear to be particularly unhelpful for performance in terms of the intensity and direction of emotions experienced, the ‘Facilitating Arousal’ profile was found to be associated with more adaptive cognitive appraisals and coping strategies. For example, athletes in this group will likely experience greater challenge and active coping, and less denial. This profile typifies an athlete who can view emotions as helpful for performance. This suggests that even in the case of what may be considered as negative emotions, it is important to consider the effect attributed to emotions on performance, because each emotion influences performance in a different way depending on its particular profile (23). For example, emotions like anxiety and anger don't always harm performance, as their energy-mobilizing properties may offer potential benefits (4). Further, a more active coping strategy may mean an athlete is able to regulate their emotions (7), thus facilitating competitive performance (1).

These results offer support for the TCTSA in that when emotions such as anxiety are viewed as facilitative, it can increase the likelihood of a challenge state as an athlete approaches competition (12). Although the present study does not measure performance as such, stress theory points to the interpretation of emotions as facilitative being an antecedent of challenge states (14), and when this is the case, better performance will likely follow (13). Mental health was also not measured in this study *per se*, but it should be noted that stress theories point to adaptive cognitive appraisals and coping as an important influence an individual's mental health (9). Considering such literature, the ‘Facilitating Arousal’ profile could be advantageous in contributing to better performance and mental health.

### Strengths and weaknesses of the study

A strength of the study was the cluster analysis approach, which allowed to combine intensity and perceptions of the role of emotions to performance. In order to obtain a broader perspective of how emotions are expressed in efforts to adapt to stressful situations, it is suggested that future studies explore the role played by other emotions [e.g., guilt, shame, relief, pride, or hope; (7)]. Another important aspect would be adopting longitudinal designs and the collection of idiosyncratic data to broaden knowledge of how athletes deal with the same stressful situation over time (32). A limitation of this study was the absence of a performance measure. Not only could future studies address this cross-sectionally, but including a performance measure as part of an intervention to develop adaptive emotion direction could allow us to further understand the causal effects of emotion direction on performance. Future studies could also capture athletes’ self-talk to gain a greater understanding of their cognitive appraisals, emotions and coping strategies in the moment (22).

### Applied implications

In terms of practical implications, these results highlight the need for psychology professionals to develop interventions that foster adaptive emotional experiences (8). For example, given the known associations between stress mindset and proactive coping (33) and that stress mindset interventions can reduce athletes’ negative affect (34), practitioners may wish to consider cultivating more adaptive views of stress to enhance affective outcomes. This could be achieved through education to highlight the adaptive components of stress or by including imagery training that aims to reinterpret stress responses as facilitative (35). Given the social nature of sport (21), coaches should also be mindful of their dialogue when interacting with athletes due to the potential for loss-focused language to give rise to negative emotions and threat appraisals (36). For example, coaches could include reference to enhancing perceived control, self-efficacy, and approach motivation in pre-match team talks as a mechanism for cultivating challenge states (37).

## Conclusion

Emotions are essential to understanding how athletes adapt effectively to stress and both the intensity and direction should be considered (2, 9). Broadly speaking, the athletes in this study did not report particularly unhelpful emotion intensity and direction, although the ‘Facilitating Arousal’ profile was found to be the emotional profile that was most likely to be associated with adaptive cognitive appraisals and coping strategies. This profile was the cluster in which emotions, even ones typically associated as being unhelpful to performance, were consistently recognized to be facilitative for performance. Overall, this implies that athletes who are able to look beyond the intensity of an emotion and weigh up how it could be used to facilitate performance will likely experience more adaptive cognitive appraisals and coping strategies.

## Data Availability

The raw data supporting the conclusions of this article will be made available by the authors upon request.

## Footnotes

^1^Underage athletes who participated were part of A teams.
